# A Benchmark Dataset for Rat Social and Aggressive Behavior Classification

**DOI:** 10.1038/s41597-026-07888-8

**Published:** 2026-07-21

**Authors:** Xutian Chen, Guangyu Li, Zihan Zhang, Mingkun Xu, Zuoren Wang, Qianqian Shi

**Affiliations:** 1 Guangdong Institute of Intelligence Science and Technology, Zhuhai, 519031 China; 2 Institute of Neuroscience, State Key Laboratory of Neuroscience, Center for Excellence in Brain Science and Intelligence Technology, Chinese Academy of Sciences, Shanghai, 200031 China; 3 Center for Brain-Inspired Computing Research (CBICR), Department of Precision Instrument, Tsinghua University, Beijing, 100084 China

## Abstract

Social interactions are central to behavioral and systems neuroscience, yet progress in understanding their neural basis depends on reliable and scalable behavioral quantification. However, standardized datasets, transparent annotation schemes and reproducible benchmarks for social-behavior classification remain limited. Here, we present a curated video dataset of rat social interactions recorded under the resident-intruder paradigm. The dataset covers non-social, social and aggressive behaviors, with aggression-related episodes further annotated into ethologically defined fine-grained subtypes. To support automated analysis of these dynamic and contact-rich interactions, we provide a reproducible pose-based workflow that converts DeepLabCut-derived body-part trajectories into structured features describing individual movement and inter-animal spatial relationships. Using this feature representation, we benchmark representative traditional machine learning and deep learning models under a unified evaluation protocol. These benchmarks provide reference performance for both coarse-grained social behavioral classification and fine-grained aggression-related behavior recognition, while documenting differences in class-wise performance, training efficiency and model complexity. Together, this dataset and workflow provide an open resource for developing, evaluating and comparing automated methods for rat social-interaction analysis.

## Background & Summary

Conspecific social behavior is a central subject in behavioral neuroscience and ethology^1^. During these interactions, animals integrate olfactory, auditory, tactile, and visual cues to assess social context and guide behavioral responses. These processes are supported by distributed neural and neuromodulatory systems, including the medial prefrontal cortex (mPFC)^2^, anterior cingulate cortex (ACC)^3^, amygdala^4^, and hypothalamic networks^5^. Despite these insights, a detailed understanding of the neural mechanisms underlying social behavior remains limited, in part because social interactions are highly dynamic and difficult to quantify. Rapid transitions, frequent physical contact, and subtle postural changes that characterize social interactions make automated behavioral classification challenging, leaving many studies dependent on labor-intensive expert annotation, which is time-consuming and difficult to scale to large video datasets^6^. Automated and computer-assisted approaches can help reduce this annotation burden and support more efficient behavioral analysis.

These challenges motivated us to establish a curated dataset for objective, scalable and reproducible quantification of social behavior. We collected overhead (top-view) video recordings of freely interacting rat pairs in a controlled laboratory setting under the resident–intruder paradigm. We focused on rats because they are widely used in social behavior research and, in many experimental settings, display more elaborate social interaction patterns than mice^7^, making them well suited for developing standardized behavioral datasets.

The recordings were manually annotated using an ethologically defined scheme. Behaviors were first grouped into three broad categories: non-social behavior, social (affiliative) behavior, and aggressive behavior. Aggressive episodes were further annotated into seven specific classes, including chase, clinch, lateral threat, bite, upright posture, keep down, and freezing. To ensure that subsequent analyses were based on reliable information, we further curated a subset of high-quality recordings from the full collection to support downstream analysis and benchmarking.

To analyze this video dataset, we required a markerless and scalable method for converting freely interacting rat videos into quantitative behavioral features. Earlier approaches relying on attached physical markers, wearable devices, or handcrafted visual features have provided useful tools, but they can interfere with natural behavior and are often constrained by cost, occlusion, specialized hardware requirements, or limited generalizability across experimental settings^8,9,10,11^. DeepLabCut (DLC)^12,13^ is well suited to this task, as it provides markerless tracking of user-defined body parts, requires only a limited number of manual frame annotations for training, and generates pose trajectories that can be transformed into spatial and motion-derived features, thereby supporting large-scale behavioral analysis across diverse applications^14–18^.

Using DLC, we annotated key body points across multiple anatomical regions, ranging from the head to the tail, in order to capture a broad spectrum of body movements and degrees of freedom. The resulting trajectories were converted into features of rat social behavior, including movement distance, velocity, and angular relationships between key points. These features provided a unified representation for subsequent behavior classification.

For downstream classification, we followed and extended prior pose-based behavioral analysis approaches that combined animal pose trajectories with statistical or machine-learning models. Lorbach *et al*.^19^ combined DLC-based pose estimation from top-view videos with Gaussian Mixture Models (GMM), whereas subsequent studies have further applied deep learning–based models, including convolutional neural networks (CNNs) to complex multi-animal interactions^20,21^ and long short-term memory (LSTM) networks to temporal feature modeling in animal behavior datasets^22–28^. These studies guided our selection of representative models for benchmarking and highlight that different approaches differ substantially in their data requirements and in how they exploit the structure of pose-derived features.

Motivated by this diversity of analytical strategies, we benchmarked both traditional machine-learning and deep-learning models on the present dataset for systematic comparison. Specifically, we evaluated CNN^29,30^, GMM^31^, LSTM^22,23^, light gradient boosting machine (LightGBM)^32^, Random Forest (RF)^33^, and extreme gradient boosting (XGBoost)^34^ using the same DLC-derived feature representation. We note that the LSTM benchmark in this work was evaluated under the same single-frame input setting (T=1) used by all other classifiers, so it operates as a non-temporal baseline rather than a sequence model; benchmarking LSTM with longer temporal windows is left for future work using the temporal information already provided in the released dataset. Rather than advocating a single optimal model, our goal was to establish a reproducible and extensible benchmarking framework for comparing different modeling strategies on rat social behavior classification. This framework facilitates assessment of how different models, ranging from probabilistic and tree-based methods to deep neural networks, perform on pose-derived features. Collectively, the annotated dataset, curated video subset, pose-estimation workflow, and model benchmarks provide a practical foundation for scalable and reproducible social-interaction analysis in rats.

## Methods

In this study, we focused on the characterization of rat social behaviors and provided a video dataset designed to support systematic behavior analysis. Based on established ethograms^35^, rat behaviors recorded under the resident–intruder paradigm were categorized into three coarse-grained categories: non-social behavior, social (affiliative) behavior, and aggressive behavior. Aggressive interactions were further annotated into seven fine-grained subcategories: chase, clinch, lateral threat, bite, upright posture, keep down, and freezing. Detailed behavioral definitions and annotation procedures are described below.

**Fig. 1:**
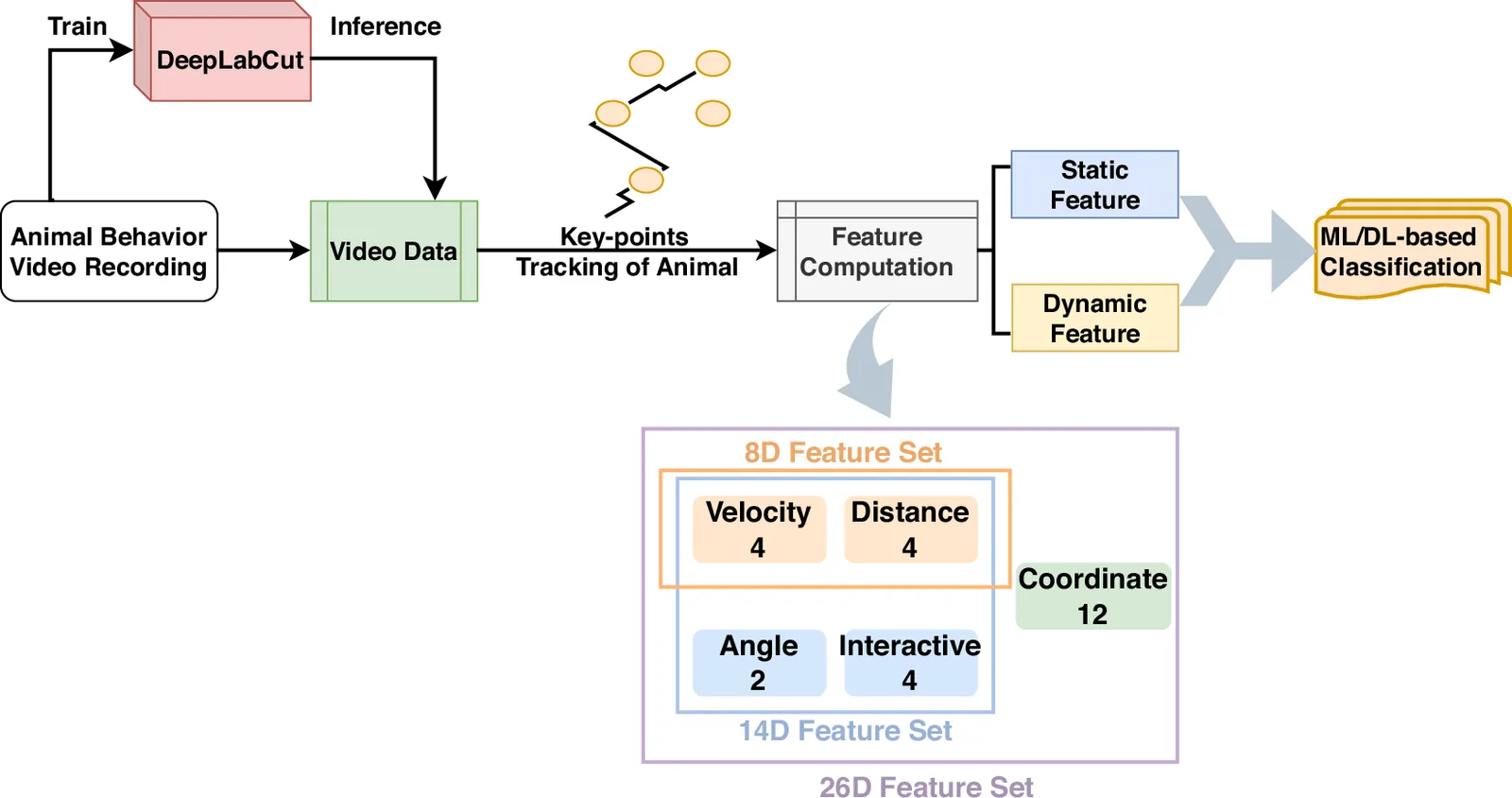
The overview of our workflow.

To support automated and reproducible analysis of the dataset, we provided a pose–based workflow as a reference implementation. In this workflow, body landmarks of the two interacting rats were first tracked from static top-view videos using DLC. The resulting keypoint trajectories were then transformed into pose-derived behavioral features that describe body position, movement, and inter-animal spatial relationships. These features were used as inputs for downstream behavior classification, as illustrated in Fig. 1.

**Fig. 2:**
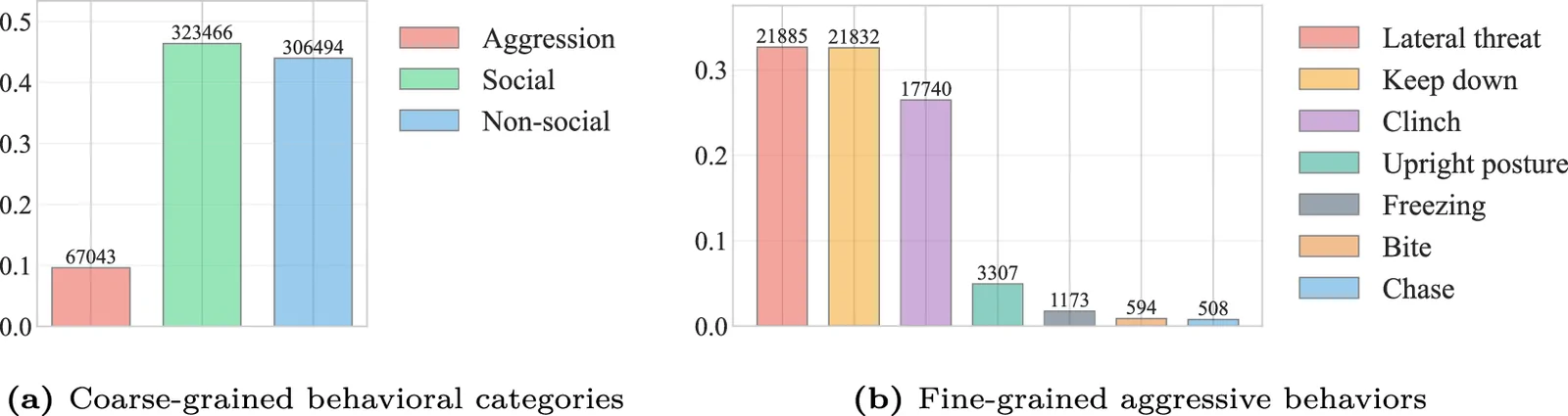
Distribution of manually annotated behavioral labels across two classification granularities.

Within this workflow, we benchmarked a set of representative modeling approaches, including traditional machine-learning and deep-learning models under a unified feature representation and evaluation protocol. The workflow therefore provides a reproducible starting point for pose-based social behavioral analysis and future methodological extensions, rather than a claim for a single optimal classifier. The distribution of annotated coarse-grained behavioral categories and fine-grained aggressive subtypes is illustrated in Fig. 2.

### Data collection

All experimental procedures were conducted in accordance with the institutional guidelines and were approved by the Animal Care and Use Committee of the Center for Excellence on Brain Science and Intelligence Technology (Institute of Neuroscience), Chinese Academy of Sciences (approval No. NA-002-2025). Euthanasia of animals was carried out strictly in accordance with the institutional guidelines, using CO_2_ to ensure animal welfare.

Behavioral experiments were performed in a dedicated behavioral testing room equipped with continuous video recording. Indoor lighting was adjusted to achieve a floor-level illumination of approximately 150 lux. Ambient environmental conditions were maintained at 25 ± 1 °C with a relative humidity of 40 ± 5% throughout all experiments. Prior to each testing session, the room was sanitized and deodorized using 75% ethanol, a disinfectant solution, and a deodorant to minimize residual olfactory cues. Rats were transferred to the testing room at least 2 hours before the experiment to allow adequate acclimation.

The dataset was derived from video recordings of multiple pairs of rats undergoing the resident–intruder test (RIT), a widely used paradigm for assessing social interaction and aggression in rodents^35^. The experimental protocol followed established procedures described in the literature. To promote territorial behavior, resident (test) male rats were housed with female cage mates in their home cages for one week before testing, during which the cages were not changed. During each test session, the resident rat’s home cage was placed in a soundproof box under video surveillance, the cage lid was replaced with a wire-mesh cover, and the female cage mate was removed. An unfamiliar intruder rat was then introduced into the cage to allow spontaneous social interaction with the resident subject.

Each interaction session was recorded continuously for 10 minutes using a fixed top-view camera at 20 frames per second and 1080p spatial resolution, yielding approximately 12,000 frames per video.

Each resident rat was tested with no more than two different intruder rats, with a minimum inter-session interval of 2 hours. After each RIT session, the behavioral room and apparatus were thoroughly cleaned again using 75% ethanol, disinfectant, and deodorant.

### Behavioral annotation

Behavioral annotations were performed manually using the Bannotator software^36^ under a single-blind protocol. Video clips were randomized and re-coded by one experimenter, and a second experimenter independently annotated the behaviors while blinded to animal identities, experimental conditions, and the original video numbering. These annotations constitute the reference behavioral labels provided with the dataset and serve as the ground truth for downstream analysis.

For each labeled event, the start and end frames as well as the assigned behavior category were recorded, yielding the per-event time intervals and the frame-level behavior labels released with the dataset.

To exclude non-experimental footage, a “base” tag was assigned to video segments recorded before the introduction of the intruder and after the termination of the test. These segments reflected manual initiation and termination of video recording and were removed prior to analysis, as they were not included in behavioral statistics.

Inter-animal interaction behaviors were annotated into three coarse-grained categories: non-social behavior, affiliative social behavior, or aggressive behavior. Non-social behavior refers to periods during which an animal exhibits neither physical contact nor directed attention toward the conspecific. Affiliative social behavior includes affiliative interactions such as social exploration, anogenital sniffing, and social grooming. Aggressive behavior comprises threat displays, pursuit, and physical confrontation, and is further subdivided into seven fine-grained action classes: chase, clinch, lateral threat, bite, upright posture, keep down, and freezing. Chase is defined as active pursuit; clinch as mutual grappling; lateral threat as a sideways intimidating display; bite as direct biting contact; upright posture as a confrontational stance on the hindlimbs; keep down as physical suppression of the opponent; and freezing as abrupt immobility during an interaction episode. These annotations follow established criteria in prior work on the resident–intruder paradigm^35^.

### DLC-based data processing

To enable pose-based analysis of the behavioral videos, we applied DeepLabCut to extract keypoint trajectories from the recorded videos.

For DLC model training, approximately 1,000 frames were sampled from a representative subset of videos in the full collection to ensure broad coverage of poses and interaction scenarios encountered in the dataset. These frames were saved as individual images for manual keypoint labeling following the standard DLC workflow^13,17^.

**Fig. 3:**
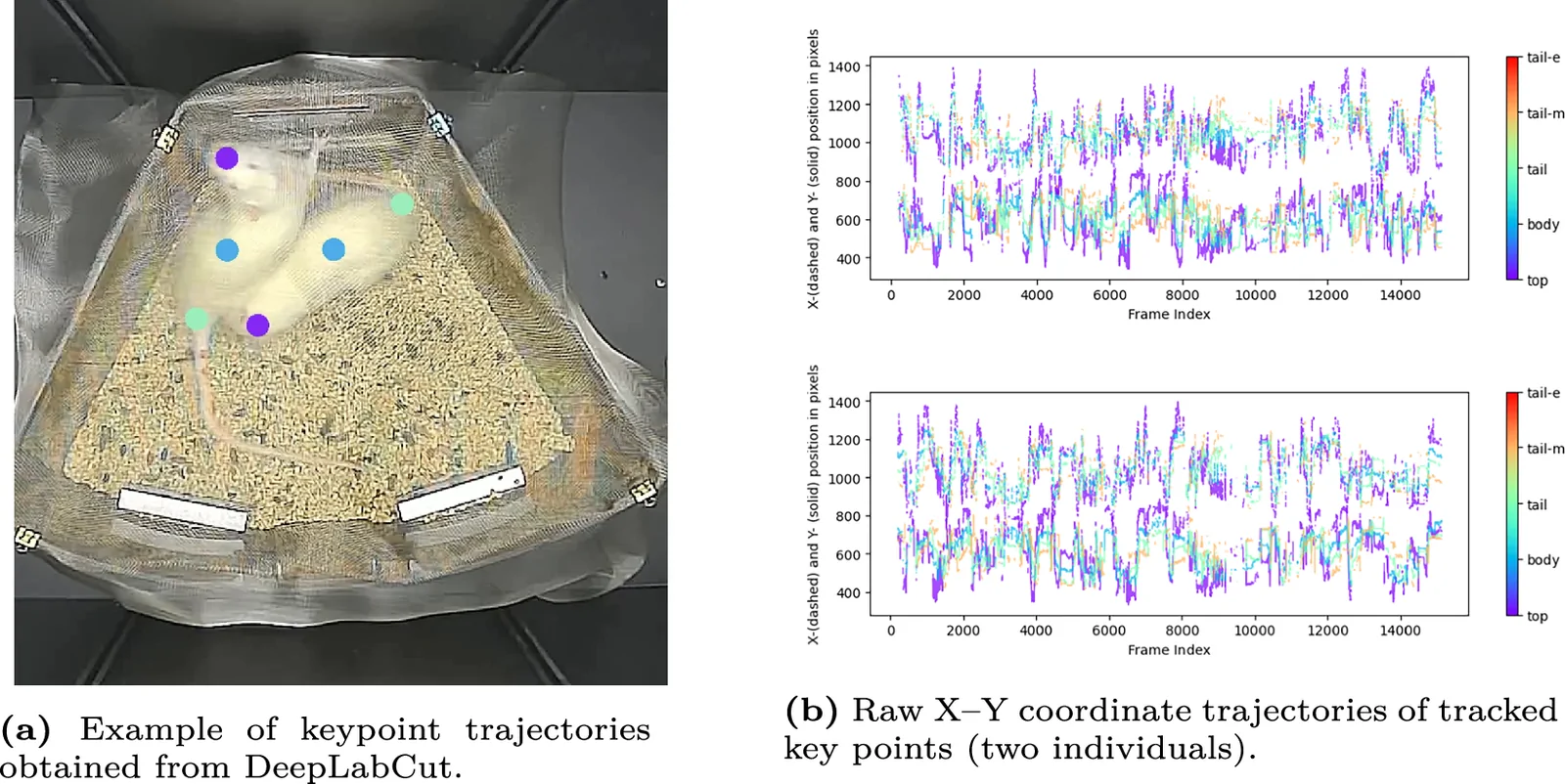
Example of keypoint tracking data obtained using DeepLabCut. (**a**) Keypoint annotations overlaid on a representative video frame. (**b**) Raw X-Y coordinate trajectories of tracked keypoints from two individuals across time.

Within DLC, the two interacting rats were assigned separate identities, denoted as subject1 and subject2. For each subject, three consistently visible top-view anatomical landmarks were manually labeled: the top of the head (top), the center of the body (body), the root of the tail (tail), as shown in Fig. 3. These manually labeled frames were used to train the DLC keypoint-tracking model.

The trained DLC model was then applied to the full set of behavioral videos to generate two-dimensional (x,y) pose trajectories for each body landmark of both rats. These pose trajectories served as the primary input for downstream feature extraction and behavior-classification analyses.

#### Feature extraction

The raw output of the DLC model consists of time series of two-dimensional coordinates representing key body landmarks of two interacting subjects (rats). To facilitate pose-based behavior classification, we derived a set of structured features from these coordinate trajectories, which are provided as part of the dataset and accompanying analysis workflow.

Extracted features were grouped into two types: static features and dynamic features. Static features include the original (x,y) coordinates of key points, pairwise distances between anatomical landmarks, and angles defined by landmark triplets. Dynamic features capture temporal changes in posture and movement, including quantities such as pointwise velocity and acceleration, computed from frame-to-frame differences of static features. The complete set of pose-derived features is summarized in Table 1. To evaluate the contribution and effectiveness of different feature groups for behavior classification, we further constructed feature subsets with increasing dimensionality, including 8-dimensional (8D), 14-dimensional (14D), and 26-dimensional (26D) configurations. The composition of these feature subsets and their impact on classification are described in the Technical Validation section.

**Table 1:** Behavioral and interaction features derived from pose-tracking keypoint trajectories for classification.

Feature	Temporal derivative	Symmetric across subjects	Type	Description
top1_speed	x	—	Velocity	Velocity of the top (head) point of subject 1
top2_speed	x	—	Velocity	Velocity of the top (head) point of subject 2
body1_speed	x	—	Velocity	Velocity of the body center of subject 1
body2_speed	x	—	Velocity	Velocity of the body center of subject 2
top_distance	—	x	Distance	Distance between the top points of both subjects
body_distance	—	x	Distance	Distance between body centers of the two subjects
top1-tail2_distance	—	x	Distance	Distance from the head of the first rat to the tail of the other
top2-tail1_distance	—	x	Distance	Distance from the tail of the second rat to the head of the other
angle_top1_tail1	—	x	Angle	Tail orientation angle of the first rat
angle_top2_tail2	—	x	Angle	Tail orientation angle of the second rat
top1_x / top1_y	—	—	Coordinate	Coordinates of the head of subject 1
body1_x / body1_y	—	—	Coordinate	Coordinates of body center of subject 1
tail1_x / tail1_y	—	—	Coordinate	Coordinates of tail root of subject 1
top2_x / top2_y	—	—	Coordinate	Coordinates of the head of subject 2
body2_x / body2_y	—	—	Coordinate	Coordinates of body center of subject 2
tail2_x / tail2_y	—	—	Coordinate	Coordinates of tail root of subject 2
relative_angle	—	x	Interactive	Difference in tail orientation: (angle_top1_tail1 − angle_top2_tail2)
top_speed_ratio	x	x	Interactive	A smoothed speed ratio defined as (top1_speed + 1)/(top2_speed + 1)
approach_speed	x	x	Interactive	Negative gradient of the top-to-top distance
body_speed_diff	x	x	Interactive	Absolute difference in body speed between two rats

Top-down recordings of freely interacting rats inevitably involve partial occlusions, close body contact, and occasional concealment near enclosure boundaries, which can lead to missing or unreliable keypoint detections, particularly for distal landmarks such as the tail. To improve robustness under these conditions, we incorporated multiple distance- and motion-related dynamic features that characterize relative movement and interaction patterns between animals, thereby providing complementary information beyond raw coordinates and helping to mitigate the impact of transient tracking failures.

To assess the robustness of the feature representation and the effect of feature dimensionality on model performance, we further defined three feature subsets of increasing complexity. The 8D baseline set focuses on fundamental kinematics and proximity, comprising four speed features (head/body velocity for both subjects) and four pairwise distance metrics (inter-head, inter-body, and head-to-tail distances). The 14D intermediate set expands upon this by incorporating geometric and interactive complexity, adding two angular features and four interaction-specific descriptors. Finally, the 26D full set encompasses all extracted dynamic and static features, providing the most comprehensive behavioral description. The corresponding ablation results are reported in the Technical Validation section.

Missing values arising from tracking artifacts were handled using linear interpolation, followed by forward and backward filling within each sequence to maintain temporal continuity.

#### Data splitting and input construction

To prevent information leakage across videos, data partitioning was performed strictly at the video level using a group-shuffle split (GroupShuffleSplit), with each recording assigned to exactly one of the training, validation, or test sets. Within each split, every frame was treated as an independent sample, characterized by a per-frame feature vector ${\bf{x}}\in {{\mathbb{R}}}^{F}$ (with feature dimensionality *F* = 8, 14, or 26 depending on the selected feature set). Temporal context is therefore captured implicitly through engineered velocity and inter-animal relative-motion features rather than via explicit sequence input.

All features were standardized using z-score normalization (StandardScaler), with normalization parameters estimated exclusively on the training set and subsequently applied to the validation and test sets to avoid information leakage. This pre-processing yielded a final input feature matrix ${\bf{X}}\in {{\mathbb{R}}}^{N\times F}$, where *N* is the total number of frames pooled across videos within the given split and *F* is the per-frame feature dimensionality.

#### Model-based behavioral analysis

To illustrate how the extracted pose-based features can be used for behavioral analysis, we evaluated a set of representative modeling approaches commonly applied in animal behavior studies. These models serve as reference implementations within the proposed workflow.

**CNN**. Convolutional neural networks (CNNs)^29,30^, as one of the most representative deep learning models, have demonstrated remarkable success in visual and spatiotemporal analysis tasks. Although the present study is based on pose-derived features rather than raw images, we included a CNN-based approach as a representative neural network model within the benchmark workflow.

To accommodate the non-image nature of the data, we employed a one-dimensional convolutional neural network (1D-CNN) operating on per-frame feature vectors. Our CNN is a lightweight 1D architecture: two convolutional blocks (kernel size 3) applied across the feature dimension (channel dimensions 1 → 32 → 64), each followed by BatchNorm and ReLU. The convolution captures local interactions among adjacent feature channels of a single frame rather than temporal dependencies across frames. The flattened representation is passed through *Dropout(*p=0.5*)* and a fully connected head (*fc1:* $64\times F\to 128$, *fc2:* 128→C). Model training was performed using a class-balanced cross-entropy loss and the Adam optimizer.

**GMM**. Gaussian mixture models (GMM) have been widely used for unsupervised and semi-supervised modeling of animal behavior. In previous research, Fujimori^37^ combined DeepLabCut-based pose estimation with support vector machines (SVM) to classify animal behavior in three typical situations: lying down, sitting, and eating. Similarly, Lorbach^19^ employed Gaussian Mixture Models (GMM) to recognize rat behavior across five categories. These studies validate the feasibility of probabilistic models for animal behavior recognition. In the present study, we included a GMM-based approach as a probabilistic reference model for multi-class rat behaviors under more complex interaction scenarios.

For this implementation, each frame’s feature vector served as an independent training sample, with the per-frame behavioral label as the supervisory signal. All samples were standardized using z-score normalization to eliminate scale differences across features prior to model fitting.

For classification, a separate GMM was trained for each behavior category. Each model consisted of multiple Gaussian components with full covariance matrices to capture the variability of the feature distributions. During inference, the log-likelihood of an input sample under each category-specific GMM was computed, and the category associated with the highest likelihood was selected as the predicted label, yielding a generative multi-class classification scheme.

We implemented the GMM using the GaussianMixture class from scikit-learn, with the number of components set to 10, covariance_type set to full, and n_init set to 10 to ensure robust maximum-likelihood estimation. This configuration models the feature distribution using mixtures of full-covariance Gaussians and produces posterior probabilities over mixture components during inference.

**LSTM**. Long short-term memory (LSTM) networks are widely used neural architectures for modeling sequential data. Within the present benchmark, we included an LSTM as a representative recurrent deep baseline. In this implementation, each input sample is a single per-frame feature vector (effective sequence length *T* = 1); the LSTM therefore serves as a drop-in non-temporal deep baseline rather than a model of temporal dynamics. Explicit sequence-level extensions are left for future work.

Our model uses a bidirectional LSTM with hidden_size of 128 and 2 layers, with dropout of 0.3 applied between layers. The forward and backward last hidden states are concatenated and passed to a fully connected classifier head ($256\to 64\to C$) with ReLU activation and dropout.

Input features were standardized using z-score normalization based on statistics computed from the training set. Model training was conducted using a class-balanced cross-entropy loss and the Adam optimizer. To improve training stability, we applied standard optimization strategies including learning-rate scheduling, early stopping, and gradient clipping.

**LightGBM**. Light gradient boosting machines (LightGBM)^32^ is a distributed gradient boosting framework based on decision tree algorithms. The original intention of the design was to provide a fast, efficient, low memory, high accuracy tool that supports parallel and large-scale data processing. LightGBM can reduce the use of data memory, reduce the cost of communication, improve the efficiency of multi-machine parallel, and achieve linear acceleration in data computing.

For multi-class behavior classification, we adopted the native multi-class objective provided by LightGBM, with the number of classes set to *C*. The model was trained using the following hyperparameter configuration: num_leaves = 31, learning_rate = 0.1, feature_fraction = 0.8, bagging_fraction = 0.8, bagging_freq = 5, and n_jobs = −1. These parameters were chosen to provide a stable and commonly used reference setting rather than to optimize task-specific performance.

**Random Forest**. Random Forest^33^ is an ensemble learning method that combines multiple decision trees trained on bootstrap-resampled data and random subsets of features. Within the benchmarking framework, we included Random Forest as a representative bagging-based tree ensemble model.

In this study, the Random Forest model was configured with n_estimators = 100, max_depth = 10, and min_samples_split = 5. To account for class imbalance, class_weight was set to balanced. Parallel training was enabled using n_jobs = −1. All input features were standardized prior to model fitting, and final predictions were obtained by majority voting across trees.

**XGBoost**. Extreme gradient boosting (XGBoost)^34^ is a widely used tree-based ensemble learning framework for structured data analysis. Within the benchmarking framework, we included XGBoost as a representative gradient boosting approach for modeling pose-derived behavioral features.

For multi-class behavior classification, we employed the XGBClassifier with a multi-class objective (multi:softmax or multi:softprob). The model was configured using commonly adopted hyperparameters: max_depth = 6, learning_rate = 0.1, subsample = 0.8, colsample_bytree = 0.8, and reg_lambda = 1.0. Input features were standardized using z-score normalization. XGBoost was trained without explicit per-class reweighting (no scale_pos_weight or sample_weight); this serves as an unweighted gradient-boosting reference. See Table 2 for a per-model summary of class-imbalance handling.

**Table 2:** Benchmark input parity across the six benchmarked models.

Method	Family	Input type	Input shape	Temporal length *T*	Feature dim *F*
CNN	Deep	sequence (T=1)	(B,1,F)	1	26
LSTM	Deep	sequence (T=1)	(B,1,F)	1	26
LightGBM	Tree	flattened	(B,F)	1	26
RandomForest	Tree	flattened	(B,F)	1	26
XGBoost	Tree	flattened	(B,F)	1	26
GMM	Probabilistic	flattened	(B,F)	1	26

All methods receive the same per-frame feature vector (F=26 in the main results) with no additional summary statistics. The temporal length T=1 for every model; temporal information is captured implicitly through engineered velocity and inter-animal relative-motion features rather than via explicit sequence input. Deep models receive the input as (B,1,F) and tree/probabilistic models as (B,F); this is a wrapper-level shape convention and does not introduce additional temporal information.

#### Implementation details and hyperparameter tuning

All deep learning models (CNN, LSTM) were implemented using PyTorch. Optimization was performed using the Adam optimizer with an initial learning rate of $1\times {10}^{-3}$, weight decay of $1\times {10}^{-4}$ for regularization, and a batch size of 256. Models were trained for a maximum of 50 epochs, utilizing early stopping with a patience of 10 epochs for CNN and 15 epochs for LSTM, monitoring validation accuracy to prevent overfitting.

For traditional machine learning baselines (RF, XGBoost, LightGBM, and GMM), we utilized Scikit-learn and their respective Python libraries. Hyperparameters were set to commonly used reference values rather than task-specific optima, to provide stable baselines under a unified protocol. Class-imbalance handling differs across models and is reported transparently: CNN and LSTM use balanced class weights in the cross-entropy loss; RandomForest uses class_weight='balanced'; LightGBM uses is_unbalance=True; XGBoost and GMM are reported without per-class reweighting (see Table 2). No data augmentation was applied to the feature vectors. Each (model, feature set) configuration was repeated with 5 random seeds controlling weight initialization and mini-batch ordering; results are reported as mean ± standard deviation across these runs, as defined in the Evaluation Protocol below. All benchmark experiments were conducted on a server equipped with an NVIDIA GeForce RTX 2080Ti GPU using the full collection of 58 video recordings captured by our team.

#### Evaluation protocol

To ensure a fair and reproducible comparison across model families, all benchmark experiments share a single evaluation protocol covering (i) input parity, (ii) data splitting, (iii) the unit of repetition (“run”) and what is held fixed versus randomized, and (iv) aggregation and stability metrics. These choices are summarized in Table 2 and detailed below.

**Input parity**. All six benchmarked models (CNN, LSTM, LightGBM, RandomForest, XGBoost, GMM) receive the same per-frame feature vector ${\bf{x}}\in {{\mathbb{R}}}^{F}$ (*F* = 26 in the main results, with *F* = 8 and *F* = 14 used in the feature-dimensionality ablation). The temporal length is *T* = 1 for every model: temporal information is captured implicitly through engineered velocity and inter-animal relative-motion features rather than via explicit sequence input. No additional summary statistics, sliding-window aggregation, or hand-crafted temporal features are appended for any model. Deep models receive the input as (B,1,F) and tree/probabilistic models as (B,F); this difference is a wrapper-level shape convention and does not introduce additional information.

**Data splitting**. A single video-level random partition was generated with GroupShuffleSplit (group=video_id, random_state=split_seed, 70/15/15 ratio for train/validation/test). The same partition is used by every model and every random seed, so that across-model comparisons are made on identical test frames. Class imbalance is preserved as it occurs in the data; per-model class-imbalance handling is summarized in Table 2 and reported transparently rather than equalized across models.

**Definition of a “run.”** We define a single *run* as one (split, speed) configuration. The data split is held fixed across all runs (so that every reported number is computed on the same test frames), while five random seeds $\{{s}_{1},\ldots ,{s}_{5}\}$ are applied to every model to control: (a) weight initialization for the neural models, (b) mini-batch ordering for the neural models, and (c) bootstrap sampling and feature subsampling for the tree-based models. Each (model, feature set) configuration is therefore evaluated under 1 split × 5 seeds = 5 runs, and all reported point estimates and dispersion summaries are computed across these five runs.

**Performance metrics**. For each model, two standard metrics are computed on the test partition for every run: accuracy and weighted F1-score. Accuracy refers to the average classification accuracy across all behavioral categories. The F1-score, defined as the harmonic mean of precision (the proportion of true positives among all predicted positives) and recall (the proportion of true positives among all actual instances), provides a balanced assessment of classification capability, particularly for minority behavior classes with limited sample sizes. In addition, per-class accuracy is reported to assess robustness to class imbalance.

**Aggregation and stability reporting**. We report the mean and standard deviation of accuracy and weighted F1 across the five runs (mean ± SD, *n* = 5). Per-class F1 scores are reported as the mean across the five runs. Stability across runs is summarized by the coefficient of variation

1 $${\rm{CV}}=\frac{\sigma }{\mu }$$

of accuracy and weighted F1 (Fig. 6c, f), where *σ* denotes the standard deviation of the performance metric and *μ* denotes the corresponding mean across the five runs; lower CV values indicate greater run-to-run stability. All tables and figures derived from the benchmark experiments therefore report *n* = 5 runs unless otherwise noted in the caption.

#### Inter-annotator reliability protocol

To quantitatively assess the consistency of the annotation protocol, four complete video recordings (v1, v10, v22, v23) were sampled from an independent 24-video holdout pool that was newly recorded under the same protocol as the main dataset but reserved exclusively for validation and never used for model training. These recordings were independently re-annotated by a second experimenter who was blinded to animal identities, experimental conditions, and the original video numbering. The second annotator used the same ethogram and Bannotator label dictionary applied throughout the dataset.

Inter-rater agreement was assessed at two hierarchical levels: a *coarse* level (social, non-social, aggression, with fine aggression subtypes collapsed into the parent class) and a *fine* level conditional on both annotators assigning the frame to an aggression subtype, evaluating agreement among the seven subtypes (chase, clinch, lateral threat, bite, upright posture, keep down, freezing). Frames labeled as *base* by either annotator were excluded. Cohen’s *κ* was computed using the canonical formulation (scikit-learn cohen_kappa_score). 95% confidence intervals were obtained by 500-iteration non-parametric bootstrap (seed = 42), and per-class *κ* was derived via one-vs-rest binarization.

#### DLC tracking validation protocol

To quantitatively validate the DLC pose estimates that underlie all downstream behavioral features, we evaluated the same pre-trained DLC model used to generate the released features on a dedicated held-out validation set. Because the recording setup is highly homogeneous across the entire dataset (identical top-view camera, fixed illumination, fixed arena geometry, 20 fps acquisition, and a matched animal cohort), the metrics measured on this holdout subset are representative of the tracking quality experienced by the downstream behavior-classification pipeline.

Two additional resident–intruder videos (20 min total at 1920 × 1080 pixels) were recorded under exactly the same protocol as the main dataset. These recordings were curated to contain extended segments of three challenging conditions: sustained *overlap* between the two animals (frames in which the two bodies share spatial extent and a top-view annotator cannot cleanly separate them), *fast motion* (rapid translational movement of one or both animals), and *normal* (non-overlapping, slow-motion) periods. Approximately 3,000 frames were sampled across the two videos and manually digitized to provide ground-truth coordinates for three anatomical keypoints per animal (Head, Body, Tail). The ground-truth digitization was performed by a trained experimenter blinded to the DLC predictions, using the same anatomical conventions applied during DLC model training.

Tracking quality was quantified using four complementary metrics: (i) per-keypoint pixel RMSE (Euclidean distance between DLC predictions and the digitized ground truth); (ii) Percentage of Correct Keypoints (PCK) at error budgets of 40 px and 60 px, following the multi-animal pose-tracking convention^21^; (iii) the percentage of frames whose DLC likelihood is at or above the operating threshold of 0.6, and the mean DLC likelihood, computed per scenario and per keypoint; and (iv) per-keypoint missingness, defined as the percentage of evaluated frames whose DLC likelihood falls below 0.6. In addition, a frame-level identity-swap audit was performed on the validation set; frames affected by a full identity swap (in which all keypoints of both animals are inverted simultaneously) were corrected by a deterministic post-processing rule based on inter-frame trajectory continuity (cross-frame Euclidean-distance–minimizing assignment) prior to RMSE and PCK computation, so that the reported pose-localization metrics reflect the swap-corrected tracks rather than raw DLC output.

## Data Records

### Dataset

**Fig. 4:**
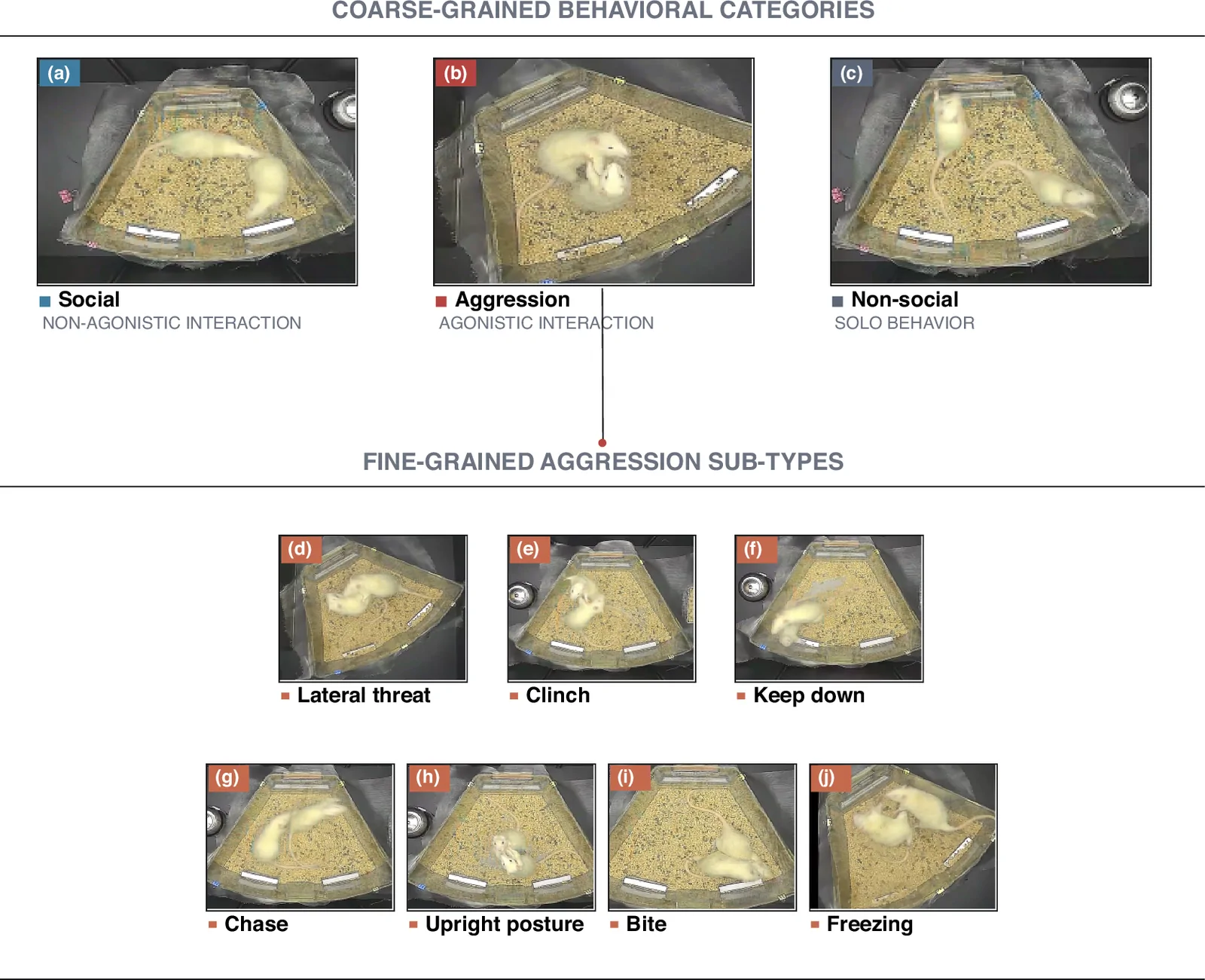
Hierarchical illustration of annotated rat social interaction behaviors. Coarse-grained behavioral categories include social, aggression, and non-social. The aggression category is further subdivided into seven fine-grained aggressive behavior subtypes, each illustrated using representative image examples.

The complete dataset described in this work is publicly available on Figshare without access restrictions^38^. The dataset is organized into several main folders, each corresponding to one resident–intruder test session. Each folder contains a top-view video recording of social interactions between two rats under controlled laboratory conditions, together with the corresponding behavioral annotation files in.txt or.mat format. Each annotation file corresponds to a single video and includes coarse-grained behavioral labels, including non-social, social, and aggressive behavior. Aggressive episodes are further labeled into fine-grained subcategories, including chase, clinch, lateral threat, bite, upright posture, keep down, and freezing, as shown in Fig. 4. These annotations are provided as either frame-level labels or temporal event intervals, supporting both frame-wise behavioral analysis and event-based quantification.

In addition to the raw videos and behavioral annotations, we provide pose-derived feature files generated from DLC keypoint trajectories. These files contain structured behavioral features, including pairwise distances, velocities, accelerations, and angular relationships between body landmarks of the two interacting rats. These features were used as inputs for the behavior-classification benchmarks and can support downstream pose-based behavioral analysis.

Overall, this dataset offers a structured resource integrating raw video recordings, manual annotations, keypoint trajectories, and derived features. These data facilitate the exploration and development of automated methods for analyzing social and aggressive behaviors in laboratory rats.

## Technical Validation

We validated three aspects of the dataset and benchmark: inter-annotator agreement of the behavioral labels, DLC tracking quality on a manually digitized holdout set, and downstream classification performance across the 26-dimensional feature set and three benchmarked model families. The protocols underlying each validation are described in the Methods section; the results are reported below.

### Inter-annotator reliability

Annotator agreement was quantified by independent re-annotation of four videos from the 24-video holdout pool, scored with Cohen’s *κ* at both the coarse and fine levels (see Methods, *Inter-annotator reliability protocol*).

Pooled across the four videos, coarse-level agreement was κ=0.931 (95% CI 0.928–0.934, N=48,000 frames) and fine-level agreement was κ=0.887 (95% CI 0.880–0.893, N=10,674 frames), both falling in the “almost perfect” range of the Landis and Koch (1977)^39^ interpretation scale. Per-class coarse *κ* ranged from 0.906 (social) to 0.949 (non-social), and per-video coarse *κ* was ≥0.85 for all four videos (v1=0.942, v10=0.985, v22=0.858, v23=0.928), confirming that the pooled result is not driven by any single session. At the fine level, six of the seven aggression subtypes reached almost-perfect agreement (freezing 0.985, keep down 0.934, bite 0.915, lateral threat 0.880, clinch 0.869, upright posture 0.827); only the *chase* subtype reached κ=0.426 (moderate), reflecting an unresolved boundary in the operational definition of chase relative to clinch and lateral threat in the literature rather than annotator error. Full reproducible scripts, the merged annotation workbook, and detailed confusion matrices are provided in the Supplementary Materials.

### DLC tracking validation

Tracking quality was evaluated on a manually digitized held-out set using four complementary metrics—pixel RMSE, PCK at two error budgets, per-scenario likelihood-threshold statistics, and per-keypoint missingness—together with a frame-level identity-swap audit (see Methods, *DLC tracking validation protocol*). Each metric is summarized in turn below.

Table 4 reports the per-keypoint pixel RMSE on the two validation pairs. The combined overall RMSE is 49.56 px (Head 65.39, Body 40.95, Tail 41.99 pixels on a 1920 × 1080 frame), with the Body and Tail keypoints—which dominate the dyadic features used downstream—both at or below 42 px. The per-frame RMSE distribution (Table 5) is right-skewed, with a median of approximately 14 px and a mean of 30–37 px.

To complement the absolute-error perspective of pixel RMSE, we further report the PCK at two error budgets, 40 px and 60 px (Table 3). These budgets correspond to approximately 14% and 20% of the rat body length on the 1920 × 1080 frames used here.

**Table 3:** Percentage of correct keypoints on the validation set at two error thresholds, computed after full-swap correction.

Threshold	Validation pair	Head	Body	Tail	Overall
40 px	Pair 1 (video 22)	60.81%	73.46%	78.48%	70.71%
	Pair 2 (video 23)	49.52%	67.34%	68.66%	62.09%
	**Combined**	55.23%	70.13%	73.52%	**66.27%**
60 px	Pair 1 (video 22)	64.11%	76.05%	80.04%	73.21%
	Pair 2 (video 23)	51.70%	68.81%	68.89%	63.41%
	**Combined**	57.98%	72.11%	74.40%	**68.16%**

At 40 px, the combined overall PCK is 66.27%; at 60 px, it rises to 68.16%. Tail keypoints are the most reliably localized at both thresholds (73.52% and 74.40%), and Head keypoints the least (55.23% and 57.98%).

**Table 4:** Pixel RMSE of DLC pose estimates on the validation set, broken down by recording pair and keypoint.

Validation pair	Head (px)	Body (px)	Tail (px)	Overall (px)	Coord. pairs
Pair 1 (video 22)	61.93	35.74	34.03	44.98	13,088
Pair 2 (video 23)	69.43	45.30	49.37	54.07	12,140
Combined	65.39	40.95	41.99	**49.56**	25,228

RMSE is computed after full-swap correction.

Relaxing the threshold from 40 px to 60 px only adds 1.89 percentage points. This small gain indicates a bimodal per-frame error distribution. The vast majority of frames already fall within the 40 px budget, consistent with the approximately 14 px median per-frame error reported in Table 5. A residual minority of frames—concentrated in overlap segments where the two animals are physically inseparable from a top-view angle—exhibit much larger errors that are not recoverable by simply enlarging the threshold.

**Table 5:** Per-frame RMSE distribution (pixels) on the validation set, after full-swap correction.

Validation pair	Min	Q1	Median	Q3	Max	Mean	*N* frames
Pair 1 (video 22)	1.45	10.83	14.52	34.86	213.35	29.71	1,416
Pair 2 (video 23)	0.90	9.94	13.74	50.35	268.31	36.71	1,545

Table 6 reports the per-scenario, per-keypoint confidence statistics on the validation set. Three patterns emerge.

- In *normal* scenes, Tail-keypoint confidence is above threshold in more than 87% of frames in both videos. Body-keypoint confidence is above threshold in more than 77% of frames. The mean Tail confidence remains above 0.73.
- *Overlap* scenes are the dominant cause of low-likelihood Head predictions. The mean Head confidence drops to 0.375 in video 23 overlap. This is biologically expected, as the two animals’ heads frequently obscure each other from a top-view angle.
- *Overlap* and *fast motion* scenes together account for approximately 22% of frames overall, while the remaining approximately 78% are tracked with high confidence. Tracking failures are therefore concentrated in a small minority of frames in which the two animals are physically inseparable from above, rather than being distributed uniformly across the dataset.

**Table 6:** Per-scenario and per-keypoint confidence statistics on the validation set.

Video / Scenario	Frames	% of video	Head ≥ thr	Body ≥ thr	Tail ≥ thr	Head conf.	Body conf.	Tail conf.
Video 22 – overlap	226	15.75%	59.60%	43.50%	84.46%	0.519	0.538	0.698
Video 22 – fast motion	213	14.84%	56.51%	38.46%	83.43%	0.506	0.506	0.695
Video 22 – normal	996	69.41%	75.63%	77.55%	87.82%	0.627	0.640	0.732
Video 22 – overall	1,435	100.00%	70.71%	67.30%	86.73%	0.595	0.607	0.722
Video 23 – overlap	226	13.87%	36.25%	76.25%	93.75%	0.375	0.629	0.764
Video 23 – fast motion	6	0.37%	50.00%	50.00%	100.00%	0.517	0.570	0.758
Video 23 – normal	1,397	85.76%	69.76%	80.75%	90.03%	0.575	0.642	0.740
Video 23 – overall	1,629	100.00%	65.36%	80.07%	90.54%	0.549	0.640	0.743
Combined – overlap	452	14.75%	48.52%	59.05%	88.87%	0.451	0.581	0.730
Combined – fast motion	219	7.15%	56.36%	38.73%	83.82%	0.506	0.508	0.696
Combined – normal	2,393	78.10%	72.42%	79.31%	89.03%	0.599	0.641	0.736
Combined – overall	3,064	100.00%	68.03%	73.70%	88.64%	0.572	0.624	0.733

The “≥ thr” columns report the percentage of frames whose DLC likelihood is ≥0.6; the “mean conf.” columns report the mean DLC likelihood across all retained frames.

Per-keypoint missingness is summarized in Table 7 (percentage of evaluated frames in which the DLC likelihood falls below the operating threshold of 0.6). The combined overall missingness is 23.51%. Consistent with the confidence breakdown in Table 6, missingness is dominated by the Head keypoint, while Body and Tail keypoints show substantially lower rates. Missing values are handled in the downstream pipeline by linear interpolation followed by forward/backward filling within each video, as described in the Methods.

**Table 7:** Per-keypoint missingness rate on the validation set, reported as (missing frames) / (expected frames).

Video	Head	Body	Tail	Overall
Video 22	26.92%	14.66%	12.16%	18.09%
Video 23	39.62%	23.89%	22.99%	28.61%
**Combined**	**33.20%**	**19.68%**	**17.64%**	**23.51%**

Frames are counted as missing when the DLC likelihood is below 0.6.

Finally, multi-animal DLC predictions can include identity swaps, in which the two animals’ tracked coordinates are reassigned to the wrong tracks. We performed a frame-level identity-swap audit on the validation set (Table 8). The raw swap rate is 37.3% of evaluated frames, which is within the upper range reported for close-contact dyadic recordings in the multi-animal pose literature^21^. Importantly, the majority of swap frames are *full swaps* (945 of 1,285, approximately 73.5% of swap events), in which all three keypoints of both animals are inverted simultaneously. Full swaps are the most easily corrected type of error: a simple post-processing rule based on inter-frame trajectory continuity (assignments that minimize cross-frame Euclidean distance between consecutive predicted positions) detects and reverses them deterministically; this correction was applied before the RMSE values in Table 4 were computed. The residual *partial swaps* (9.9% of evaluated frames) arise almost entirely from a single visual condition—when the two animals’ Head keypoints (or, less commonly, their Tail keypoints) are within a few pixels of each other, the network occasionally assigns one keypoint to the wrong animal while the rest of the body remains correctly tracked. Partial swaps cannot be deterministically corrected by the trajectory-continuity rule and may introduce noise in downstream pair-level features such as inter-animal distances, relative angles, and relative speeds. Such errors may affect downstream analysis, particularly for close-contact aggressive behaviors in which the two animals are spatially close or partially overlapping; this may partly explain why aggressive interaction categories such as chase and lateral threat are more challenging to classify. Both the raw DLC outputs and the swap-corrected tracks are released alongside the dataset, allowing downstream users to choose either version depending on whether their analysis is identity-aware.

**Table 8:** Identity-swap statistics on the validation set.

Statistic	Value
Frames evaluated	3,441
Coordinate pairs evaluated	30,278
Frames with any identity swap	1,285 (37.3% of evaluated frames)
Full swap (all keypoints inverted)	945 (73.5% of identity swaps)
Partial swap (subset of keypoints inverted)	340 (26.5% of identity swaps)

Taken together, the validation supports the use of the released DLC tracks for downstream behavioral classification. The raw DLC outputs, the swap-corrected tracks, and the manually digitized ground-truth coordinates are all released as part of the dataset to support independent re-evaluation and downstream extensions.

### Feature dimensionality

The three feature subsets defined in the Methods (8D, 14D, and 26D; see Methods, Feature Extraction) were compared on both classification tasks. Differences in performance across the three dimensionalities are relatively small for coarse-grained social behavior classification (Table 9), but a clearer advantage of higher-dimensional features is observed for fine-grained aggressive behavior classification (Table 10). In particular, the 26D feature representation achieves the best or near-best performance across most evaluated models, including the top-performing single model.

**Table 9:** Classification performance for coarse-grained (social interaction) behavioral classification using different numbers of input features (8D, 14D, and 26D).

Model	Accuracy (8D)	F1-score (8D)	Accuracy (14D)	F1-score (14D)	Accuracy (26D)	F1-score (26D)
CNN	0.655 ± 0.009	0.624 ± 0.015	0.649 ± 0.023	0.614 ± 0.032	0.638 ± 0.023	0.611 ± 0.030
GMM	0.380 ± 0.052	0.419 ± 0.044	0.362 ± 0.050	0.389 ± 0.051	0.368 ± 0.037	0.395 ± 0.034
LSTM	0.650 ± 0.009	0.623 ± 0.015	0.639 ± 0.023	0.610 ± 0.031	0.615 ± 0.020	0.598 ± 0.026
LightGBM	**0.660** ± **0.010**	**0.634** ± **0.017**	**0.654** ± **0.023**	**0.623** ± **0.031**	**0.649** ± **0.025**	**0.622** ± **0.033**
RandomForest	0.630 ± 0.012	0.620 ± 0.017	0.624 ± 0.023	0.608 ± 0.030	0.637 ± 0.024	0.618 ± 0.029
XGBoost	0.657 ± 0.011	0.633 ± 0.018	0.650 ± 0.024	0.621 ± 0.031	0.641 ± 0.028	0.618 ± 0.035

Values are reported as mean ± standard deviation.

**Table 10:** Classification performance for aggressive behavior classification using different numbers of input features (8D, 14D, and 26D).

Model	Accuracy (8D)	F1-score (8D)	Accuracy (14D)	F1-score (14D)	Accuracy (26D)	F1-score (26D)
CNN	**0.392** ± **0.044**	0.358 ± 0.050	**0.404** ± **0.026**	0.374 ± 0.032	0.393 ± 0.039	0.381 ± 0.045
GMM	0.288 ± 0.045	0.299 ± 0.027	0.217 ± 0.062	0.222 ± 0.038	0.321 ± 0.023	0.307 ± 0.018
LSTM	0.390 ± 0.045	0.359 ± 0.051	0.393 ± 0.025	0.360 ± 0.030	0.383 ± 0.030	0.375 ± 0.036
LightGBM	0.386 ± 0.040	**0.365** ± **0.044**	0.401 ± 0.021	**0.382** ± **0.025**	**0.404** ± **0.030**	**0.392** ± **0.038**
RandomForest	0.375 ± 0.041	0.360 ± 0.044	0.385 ± 0.022	0.373 ± 0.027	0.389 ± 0.033	0.372 ± 0.039
XGBoost	0.383 ± 0.041	0.363 ± 0.046	0.398 ± 0.024	0.379 ± 0.028	0.391 ± 0.031	0.380 ± 0.037

Values are reported as mean ± standard deviation.

Fine-grained aggressive behavior classification represents a more challenging and practically relevant scenario, as aggressive and defensive behaviors are typically sparse, short-lived, and highly imbalanced in naturalistic animal behavior datasets. Such imbalance is an inherent characteristic of ethological data and is difficult to eliminate through experimental design. The improved performance of higher-dimensional features suggests that the additional spatial and temporal descriptors captured by the 26D representation provide more discriminative information for characterizing subtle differences among aggressive subtypes.

Given that the performance differences among feature dimensionalities are modest for coarse-grained classification, but more pronounced for fine-grained aggressive classification, we adopt the 26D feature setting as the default configuration for subsequent analyses to better address the practical challenges of imbalanced and complex aggressive behavior recognition.

### Feature set selection

Across models, the performance differences between the 8D, 14D, and 26D feature sets are generally modest (Tables 9 and 10), indicating that the dataset supports stable classification across a range of feature complexities, particularly for interaction-related features between the two subjects. Given that DLC-based keypoint tracking may be affected by occlusion and noise during close-contact interactions, the 26-dimensional feature set was selected for subsequent analyses, as it provides a more comprehensive description of spatial and kinematic relationships.

#### Classification results

As summarized in Table 10, the baseline models (GMM, CNN, and LSTM) achieved moderate performance on both classification tasks, reflecting the challenges posed by class imbalance and fine-grained categorization. Across all evaluated models (Tables 9–12), moderate overall performance was consistently observed, indicating that the dataset contains sufficient behavioral structure to support automated recognition while remaining non-trivial.

**Fig. 5:**
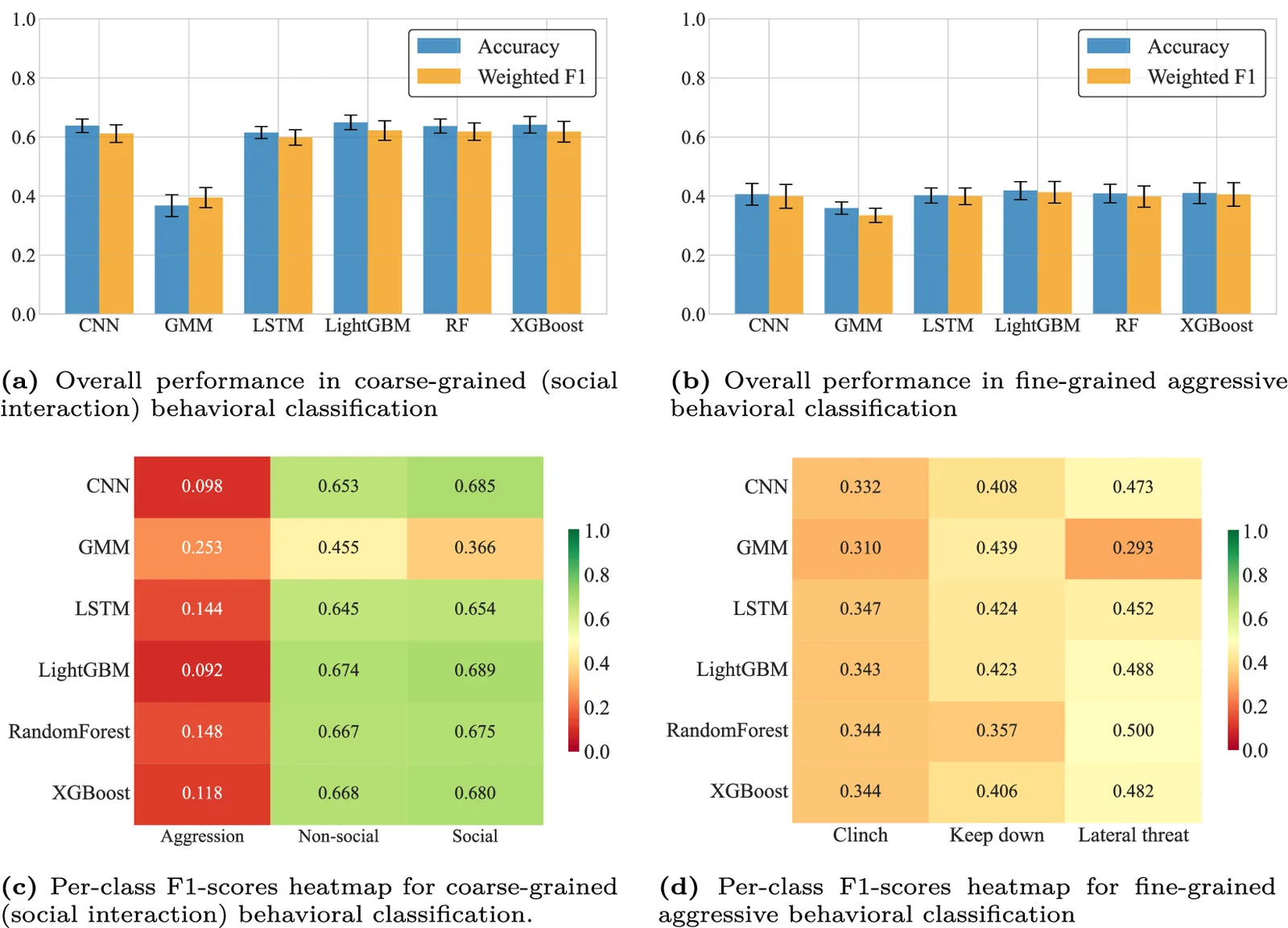
Overall comparison of classification performance, including coarse-grained (social interaction) behavioral classification results, fine-grained aggressive behavioral classification results, and corresponding per-class F1-score heatmaps.

For coarse-grained behavior classification (Table 11), higher F1-scores were consistently achieved for social and non-social behaviors than for aggression, suggesting greater variability and recognition difficulty for aggressive interactions. Similar trends were observed across both traditional machine learning and deep learning models. These patterns are further illustrated in Fig. 5, where all models show comparable accuracy and weighted F1-scores for the three-class task, indicating that coarse-grained social behavior classification can be reliably addressed under the proposed feature representation.

**Table 11:** Per-class F1 scores (%) of different models for coarse-grained behavioral classification.

Class	CNN	GMM	LSTM	LightGBM	RandomForest	XGBoost
Aggression	9.8 ± 2.6	**25.3** ± **5.3**	14.4 ± 1.6	9.2 ± 1.8	14.8 ± 1.2	11.8 ± 2.2
Non-social	65.3 ± 1.9	45.5 ± 4.3	64.5 ± 2.0	**67.4** ± **2.0**	66.7 ± 2.2	66.8 ± 2.5
Social	68.5 ± 2.4	36.6 ± 9.7	65.4 ± 2.1	**68.9** ± **2.6**	67.5 ± 2.4	68.0 ± 2.6

Values are reported as mean ± standard deviation.

For fine-grained aggression classification (Table 12), behaviors with longer duration and higher occurrence frequency, such as clinch, keep down, and lateral threat, were recognized with moderate performance across models, whereas brief and infrequent behaviors, including bite, chase, freezing, and upright posture, consistently yielded lower F1-scores. Figure 5 further confirms this pattern, showing that moderate per-class F1-scores are primarily concentrated on these representative aggressive behaviors, while others remain difficult to recognize. This disparity mainly reflects differences in behavioral duration and sample availability rather than intrinsic model limitations. Importantly, the scarcity of these short-lived aggressive and defensive behaviors is an inherent characteristic of the resident–intruder paradigm. Although challenging for supervised classification, their inclusion substantially enhances the ecological validity of the dataset and preserves rare but ethologically meaningful social signals.

**Table 12:** Per-class F1 scores (%) of different models for fine-grained aggressive behavior classification.

Class	CNN	GMM	LSTM	LightGBM	RandomForest	XGBoost
**All fine-grained aggressive behavioral classes**						
Bite	0.0 ± 0.0	0.0 ± 0.0	0.0 ± 0.0	0.2 ± 0.5	0.0 ± 0.0	0.0 ± 0.0
Chase	0.7 ± 1.0	0.6 ± 0.5	3.5 ± 4.4	3.0 ± 2.5	0.0 ± 0.0	2.5 ± 2.7
Clinch	33.9 ± 2.1	25.2 ± 10.2	34.2 ± 1.8	34.2 ± 3.1	33.4 ± 2.4	33.6 ± 3.1
Freezing	0.9 ± 2.0	0.6 ± 0.9	0.3 ± 0.5	0.0 ± 0.0	1.4 ± 2.2	0.0 ± 0.0
Keep down	40.2 ± 9.0	38.0 ± 4.0	41.7 ± 7.0	41.9 ± 6.0	34.5 ± 7.0	39.7 ± 6.4
Lateral threat	46.3 ± 4.6	34.1 ± 6.7	43.6 ± 2.7	47.5 ± 6.0	48.8 ± 6.2	46.4 ± 5.8
Upright posture	1.1 ± 1.2	2.5 ± 1.4	3.4 ± 1.9	2.1 ± 1.3	0.7 ± 0.5	1.4 ± 1.3
(lr)1-7 *Average*	17.6 ± 2.8	14.4 ± 3.4	18.1 ± 2.6	**18.4** ± **2.8**	17.0 ± 2.6	17.7 ± 2.8
**Representative high-frequency fine-grained aggressive behavioral classes**						
Clinch	35.9 ± 3.6	17.1 ± 4.5	33.6 ± 5.7	35.4 ± 4.7	33.9 ± 5.3	33.9 ± 4.5
Keep down	40.6 ± 7.5	45.8 ± 10.1	42.9 ± 7.7	41.8 ± 5.4	39.9 ± 5.2	37.6 ± 6.7
Lateral threat	43.9 ± 6.3	39.2 ± 7.2	43.6 ± 8.3	45.2 ± 7.3	45.8 ± 9.0	44.5 ± 8.4
(lr)1-7 *Average*	40.1 ± 5.8	34.0 ± 7.3	40.0 ± 7.2	**40.8** ± **5.8**	39.9 ± 6.5	38.7 ± 6.5

Values are reported as mean ± standard deviation. The highest *Average* value in each section is bolded.

In addition to predictive performance, we compared computational efficiency and model complexity across model families (Table 13). We further report the training time and model complexity of each model to characterize their computational efficiency. Tree-based models showed relatively short training times (30.2 s for LightGBM and 54.6 s for XGBoost) and moderate complexity (3,100 and 6,400 leaf nodes, respectively), whereas deep learning models required substantially longer training time (887.7 s for LSTM and 180.2 s for CNN) and larger numbers of trainable parameters (571,651 for LSTM and 220,035 for CNN). These results illustrate the differences in computational cost across different model families.

**Table 13:** Comparison of training speed and number of parameters for different models.

Model	Training Speed (s)	Number of Parameters / Leaf Nodes
LSTM	887.7	571,651
CNN	180.2	220,035
LightGBM	**30.2**	3,100
XGBoost	54.6	6,400
Random Forest	88.4	102,400
GMM	123.2	**1,134**

The lowest values (best efficiency) are highlighted in bold.

**Fig. 6:**
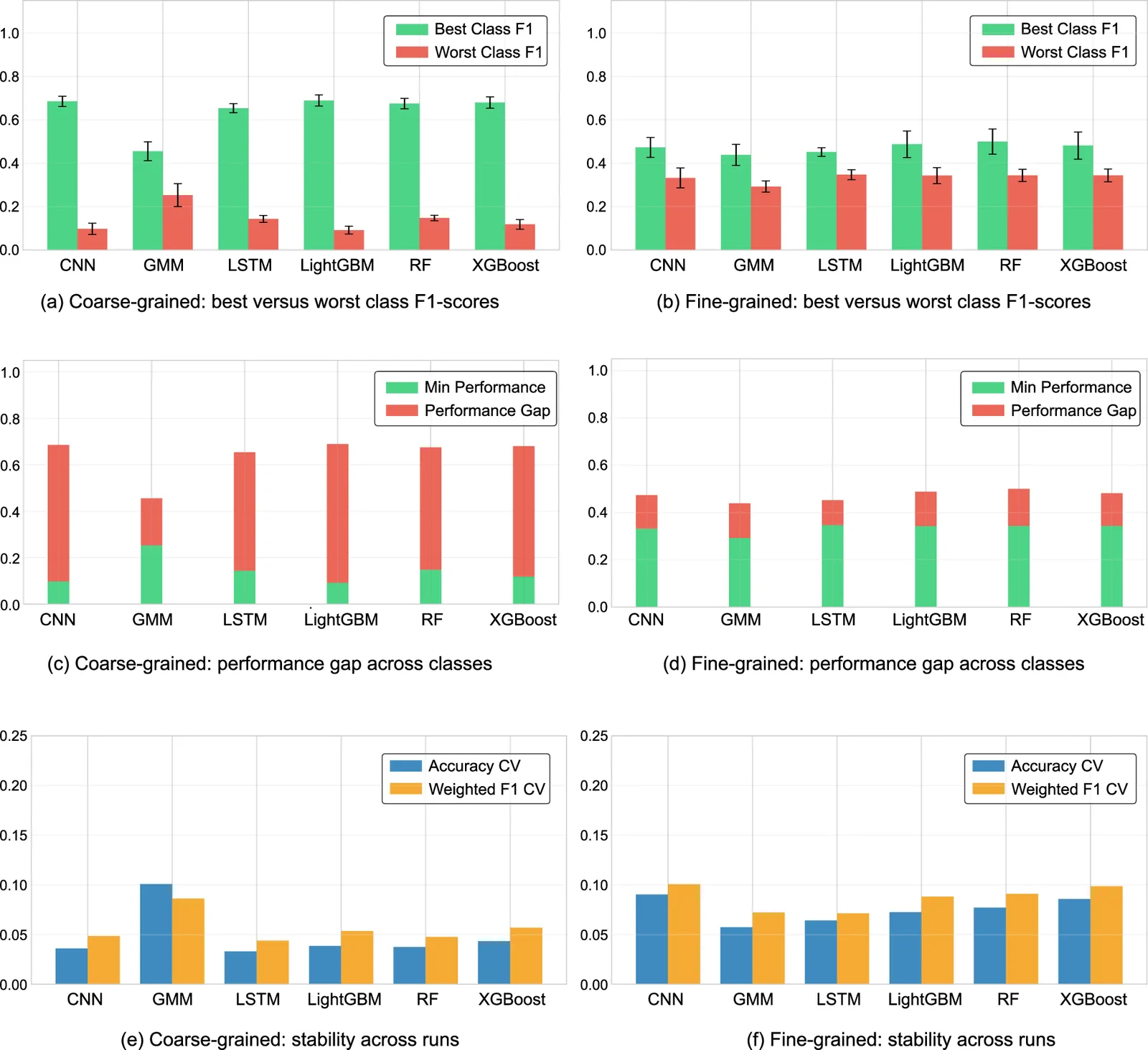
Model performance comparison organized as a 2 × 3 matrix. Columns distinguish coarse-grained (social interaction) from fine-grained aggressive behavioral classification. Rows correspond to evaluation metrics: best vs. worst class F1-scores (top), inter-class performance gap (middle), and cross-run stability (bottom).

To further assess robustness and reliability beyond overall accuracy and F1-score, we analyzed model performance from three perspectives (Fig. 6): (i) best versus worst class F1-scores, highlighting the easiest and hardest categories for each model; (ii) performance gap across classes, reflecting class-wise imbalance; and (iii) stability across runs, measured by the coefficient of variation of accuracy and weighted F1-score.

For coarse-grained social behavior classification (Fig. 6a–c), all models exhibit pronounced gaps between best and worst class F1-scores, indicating substantial differences in recognition difficulty across behavior categories. Tree-based ensemble and deep learning models show comparable patterns, and stability analysis reveals relatively low variation across repeated runs, suggesting that the results are reproducible under the current experimental setting. For fine-grained aggressive behavior classification (Fig. 6d–f), noticeable class-wise performance gaps also persist, while stability across runs remains comparable across models, indicating that fine-grained results are likewise reproducible despite increased task difficulty.

Overall, these results demonstrate that the dataset supports both coarse- and fine-grained behavioral classification, while faithfully reflecting the intrinsic complexity, imbalance, and temporal heterogeneity of naturalistic social interactions in the resident–intruder paradigm. Rather than optimizing for a single modeling approach, the reported benchmarks provide a transparent and reproducible reference that characterizes how different classes of models perform under identical feature representations and evaluation protocols.

### Ethics declarations

All experiments involving rats were conducted at the Center for Excellence in Brain Science and Intelligence Technology, Chinese Academy of Sciences. Wild-type Sprague-Dawley rats (250-350 g, 10-14 weeks old) were purchased from Shanghai, China, and were pair-housed under a standard 12-h light/dark cycle (lights on at 07:00). All experimental procedures were carried out in accordance with the institutional guidelines for animal care and use and complied with the Guidelines for the Care and Use of Laboratory Animals of the Ministry of Science and Technology of China. The study protocol was reviewed and approved by the Animal Care and Use Committee of the Center for Excellence in Brain Science and Intelligence Technology (Institute of Neuroscience), CAS (approval No. NA-002-2025). All efforts were made to minimize animal suffering throughout the experiments.

## Supplementary information

Supplmentary Movie 1

## Data Availability

The dataset used in this study is publicly available on Figshare without restrictions. It can be accessed at: https://doi.org/10.6084/m9.figshare.30393298.

## Code availability

The code used in this study can be found at Zenodo https://doi.org/10.5281/zenodo.18299934^40^. This repository contains the implementation used for evaluating rat behavior estimation. This code may be updated over time.
